# Occupational exposure to crystalline silica in a sample of the French general population

**DOI:** 10.1186/s12995-024-00402-z

**Published:** 2024-01-31

**Authors:** Pierre-Marie Wardyn, Jean-Louis Edme, Virginie de Broucker, Nathalie Cherot-Kornobis, David Ringeval, Philippe Amouyel, Annie Sobaszek, Luc Dauchet, Sébastien Hulo

**Affiliations:** 1 Univ. Lille, ULR 4483—IMPECS—IMPact de l’Environnement Chimique sur la Santé humaine, F-59000 Lille, France; 2 CHU Lille, Service de médecine du travail du personnel hospitalier, pathologies professionnelles et environnement, F-59000 Lille, France; 3 Institut Pasteur Lille, F-59000 Lille, France; 4 CHU Lille, Service des explorations fonctionnelles respiratoires, F-59000 Lille, France; 5 Univ. Lille, U1167—RID-AGE—Facteurs de risque et déterminants moléculaires des maladies liées au vieillissement, F-59000 Lille, France; 6 Inserm, U1167, F-59000 Lille, France; 7 CHU Lille, Service d’épidémiologie et de santé publique, F-59000 Lille, France

**Keywords:** Silicon dioxide, Dust, Occupational exposure, Construction industry, Metallurgy

## Abstract

**Objective:**

To describe the proportions of subjects exposed to crystalline silica and the sectors of activity concerned between 1965 and 2010 in a sample of the general French population.

**Methods:**

We included 2942 participants aged 40 to 65 years, recruited at random from electoral rolls, from the French general population in the cross-sectional ELISABET study between 2011 and 2013. The proportions of subjects exposed to crystalline silica and their sectors of activity were determined on the basis of their career history and the use of the Matgéné job-exposure matrix.

**Results:**

In the total sample, occupational exposure to crystalline silica was found for 291 subjects (9.9%) between 1965 and 2010, with a predominance of men (20.2% of exposed subjects among men (282 out of 1394) versus 0.6% among women (9 out of 1548)). The highest proportion of participants exposed to crystalline silica was reached in 1980 with 6.1% and then decreases to 4.4% in 2010. Among men, the most frequently exposed sectors of activity were manufacture of basic metals (41.5% of exposed men (117 out of 282)), specialised construction activities (23.1% of exposed men (65 out of 282)) and construction of buildings (14.2% of exposed men (40 out of 282)).

**Conclusions:**

Although the proportion of workers exposed to crystalline silica has been decreasing since the 1980s, it is still significant at least until 2010, particularly in the construction sector, and further research is needed to improve the monitoring of workers who are or have been exposed to crystalline silica.

## Introduction

Exposure to crystalline silica dust is known to cause silicosis, bronchial or lung cancer, auto-immune diseases (e.g. rheumatoid arthritis or systemic scleroderma), and non-malignant respiratory tract diseases [1, 2]. Occupational exposure to crystalline silica affects a large number of people worldwide due to its natural presence in soil, sand and rock. In the United States, it is estimated that about 2.3 million workers (i.e. around 1.5% of the labor force) were exposed in 2012 [3]. In the European Union, a 2006 estimate puts the number of potentially exposed workers at 5,300,000 [4]. In France, a study using census data and the Matgéné job-exposure matrix (JEM) estimates that 975,000 workers (i.e. about 3.8% of the active workforce) were potentially exposed in 2017, with a predominance of the construction sector followed by industry [5]. In this study, the proportion of exposed workers was also calculated using census data from 1982, 1990, 1999 and 2007, showing a decline in this proportion since 1982 with a stabilization around 4% from 1999 onwards. Although these data are the most accurate we have to date, and there is still a lack of data on the general population prior to 1982 and also a description of the sectors of activity and the proportions of subjects exposed over time. Indeed, the data currently available is limited by the census years and only provides a description of the sectors of activity for the year 2017 [5]. Our team recently highlighted the importance of monitoring silica exposure on respiratory function [6].

The objective of this study is to complement that of Delabre et al. [5] by describing the proportions of subjects exposed to crystalline silica and the sectors of activity concerned between 1965 and 2010 in a sample of the general French population.

## Methods

### Study design and population

The study participants were men and women aged 40 to 65 having participated in the ELISABET (*Enquête Littoral Souffle Air Biologie Environnement*) cross-sectional study between January 2011 and November 2013. The methodology of the ELISABET study has been described in detail elsewhere [6, 7]. Briefly, all the participants had lived in the same city or the surrounding urban area (either Lille or Dunkirk) for at least the 5 years immediately prior to inclusion. The participants were selected at random from electoral rolls, with stratification for sex, age, and city area (Lille or Dunkirk).

As the main objective of the ELISABET study was to compare the prevalence of obstructive ventilatory disorder between 2 urban areas in northern France, one with exclusively urban pollution (Lille) and the other with mixed urban and industrial pollution (Dunkirk), we excluded subjects who did not have acceptable spirometry. We also excluded subjects for whom exposure to crystalline silica could not be estimated. Following recent regulatory changes concerning data protection, information concerning the re-use of data was sent to participants of the ELISABET study, resulting in the exclusion of 89 additional subjects compared with our previous publication [6].

### Career history and exposure to crystalline silica dust

Each participant was asked by a nurse during a face-to-face interview about his first job, his latest job, the job that he had done for the longest during his career, and the various jobs that might have led to exposure to vapours, gases, fumes and/or dust. Each job was coded by combining a professional/socioprofessional code and an economic activity code. Participants were identified as exposed to crystalline silica for a given time period when the combination of their professional code and their company’s economic activity code was found in Matgéné Silice job-exposure matrix created by Santé Publique France [8]. The methodology for occupational exposure assessment has been detailed elsewhere [6].

### Statistical analysis

The proportion of subjects exposed to silica was calculated for each exposing sector of activity found in the sample and carried out between 1965 and 2010 every 5 years. The industry titles presented correspond to the Statistical Classification of Economic Activities in the European Community revision 2 (NACE) level 2 codes. The results have been detailed with NACE level 3 codes for 3 sectors of activity: manufacture of basic metals, specialised construction activities, construction of buildings. The sample analysed by year included only those aged 18 or over in the year under consideration.

### Ethical aspects

The study protocol was registered at ClinicalTrials.gov (identifier: NCT02490553) and had been approved by the local independent ethics committee (*CPP Nord Ouest IV*, Lille, France; reference: 2010-A00065-34), in compliance with the French legislation on biomedical research. All the participants gave their written, informed consent prior to inclusion in the study.

## Results

**Fig. 1:**
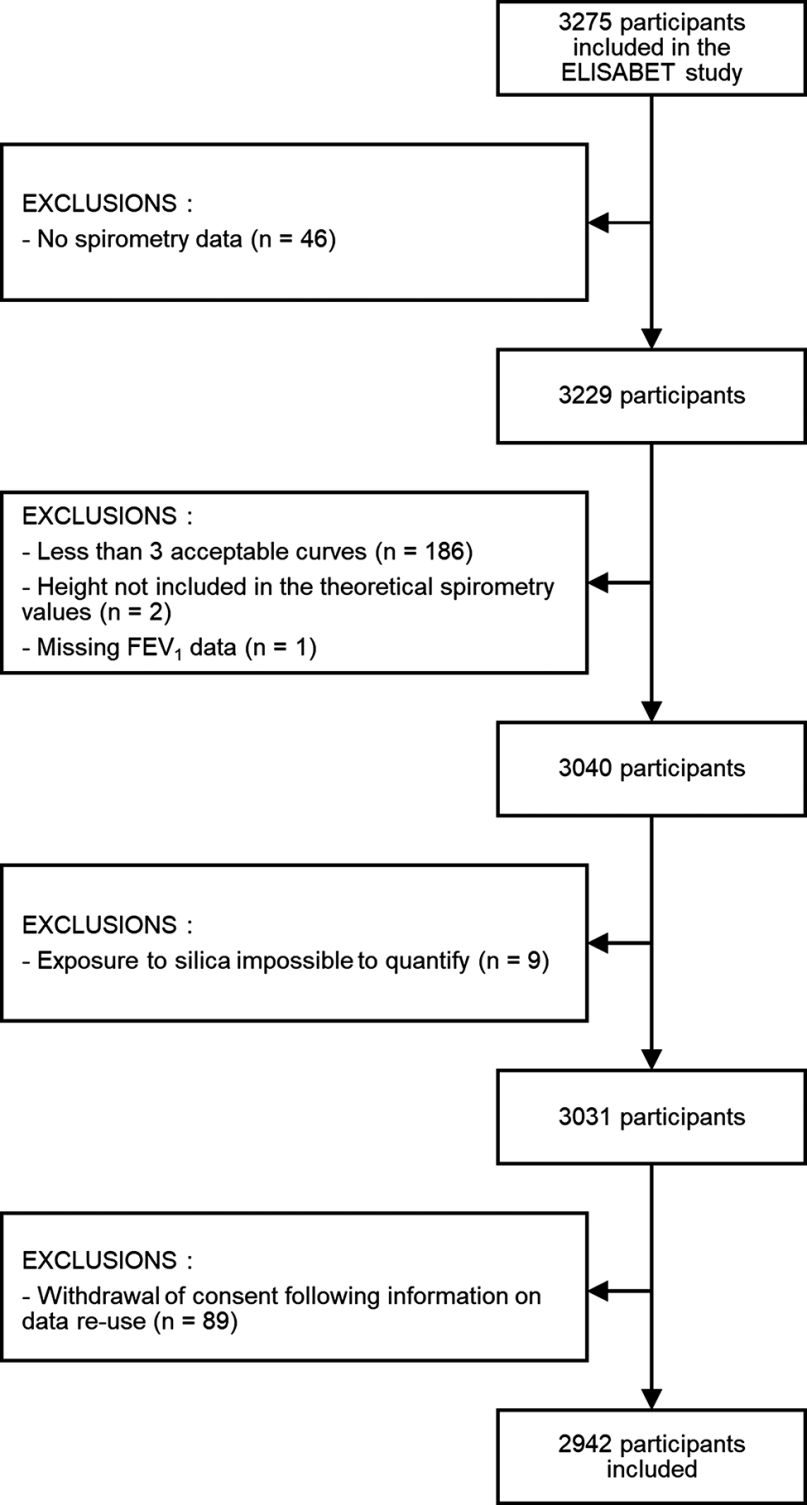
Study flow chart. FEV_1_: forced expiratory volume in the first second

In total, 2942 subjects from the ELISABET study were included (Fig. 1), of which 1394 were men (47.4%). Occupational exposure to crystalline silica was found in 291 (9.9%) of the total sample between 1965 and 2010 (101 out of 1519 (6.7%) in Lille and 190 out of 1423 (13.4%) in Dunkirk. The proportion of exposed subjects peaked in 1980 at 6.1% (117 out of 1903 active subjects) and reached 4.4% (129 out of 2942 active subjects) in 2010.

Among men, 282 subjects (20.2%) were exposed to crystalline silica between 1965 and 2010 (i.e. 96% of all exposed subjects) (Table 1). The 3 most frequently found sectors of activity were manufacture of basic metals (41.5% of exposed men (*n* = 117)), specialised construction activities (23.1% of exposed men (*n* = 65)) and construction of buildings (14.2% of exposed subjects (*n* = 40)).

**Table 1:** Proportion of workers exposed to crystalline silica and sectors of activity concerned among men

Variables	1965	1970	1975	1980	1985	1990	1995	2000	2005	2010	Overall^a^
	(*N* = 52)	(*N* = 359)	(*N* = 675)	(*N* = 933)	(*N* = 1200)	(*N* = 1394)	(*N* = 1394)	(*N* = 1394)	(*N* = 1394)	(*N* = 1394)	(*N* = 1394)
Age (Median [Q1, Q3])	18 [18, 18]	20 [19, 22]	23 [20, 26]	26 [22, 30]	29 [23, 34]	32 [26, 38]	37 [31, 43]	42 [36, 48]	47 [41, 53]	52 [46, 58]	54.1 [47.7, 59.8]
Exposure to crystalline silica	3 (5.8%)	37 (10.3%)	80 (11.9%)	113 (12.1%)	144 (12.0%)	158 (11.3%)	144 (10.3%)	148 (10.6%)	143 (10.3%)	126 (9.0%)	282 (20.23%)
Missing	-	-	*1 (0.15%)*	*2 (0.21%)*	*2 (0.17%)*	*2 (0.14%)*	*3 (0.22%)*	*2 (0.14%)*	*2 (0.14%)*	*2 (0.14%)*	-
**Sectors of activity exposed to silica exposure**											
Manufacture of basic metals	-	18 (5.01%)	37 (5.48%)	46 (4.93%)	66 (5.50%)	70 (5.02%)	61 (4.38%)	57 (4.09%)	52 (3.73%)	42 (3.01%)	117 (8.39%)
Manufacture of basic iron and steel and of ferro-alloys	*-*	*12 (3.34%)*	*28 (4.15%)*	*37 (3.97%)*	*44 (3.67%)*	*45 (3.23%)*	*40 (2.87%)*	*38 (2.73%)*	*32 (2.30%)*	*25 (1.79%)*	*76 (5.45%)*
Manufacture of tubes, pipes, hollow profiles and related fittings, of steel	*-*	*-*	*1 (0.15%)*	*1 (0.11%)*	*2 (0.17%)*	*2 (0.14%)*	*1 (0.07%)*	*1 (0.07%)*	*1 (0.07%)*	*1 (0.07%)*	*4 (0.29%)*
Manufacture of basic precious and other non-ferrous metals	*-*	*5 (1.39%)*	*6 (0.89%)*	*7 (0.75%)*	*17 (1.42%)*	*20 (1.43%)*	*17 (1.22%)*	*15 (1.08%)*	*15 (1.08%)*	*14 (1.00%)*	*29 (2.08%)*
Casting of metals	*-*	*1 (0.28%)*	*2 (0.30%)*	*1 (0.11%)*	*3 (0.25%)*	*3 (0.22%)*	*3 (0.22%)*	*3 (0.22%)*	*4 (0.29%)*	*2 (0.14%)*	*8 (0.57%)*
Specialised construction activities	1 (1.92%)	4 (1.11%)	20 (2.96%)	24 (2.57%)	24 (2.00%)	29 (2.08%)	27 (1.94%)	33 (2.37%)	32 (2.30%)	28 (2.01%)	65 (4.66%)
Demolition and site preparation	*-*	*-*	*2 (0.30%)*	*2 (0.21%)*	*2 (0.17%)*	*1 (0.07%)*	*1 (0.07%)*	*1 (0.07%)*	*1 (0.07%)*	*1 (0.07%)*	*2 (0.14%)*
Electrical, plumbing and other construction installation activities	*-*	*2 (0.56%)*	*7 (1.04%)*	*10 (1.07%)*	*9 (0.75%)*	*12 (0.86%)*	*12 (0.86%)*	*14 (1.00%)*	*13 (0.93%)*	*10 (0.72%)*	*24 (1.72%)*
Building completion and finishing	*1 (1.92%)*	*2 (0.56%)*	*3 (0.44%)*	*2 (0.21%)*	*3 (0.25%)*	*5 (0.36%)*	*3 (0.22%)*	*5 (0.36%)*	*6 (0.43%)*	*7 (0.50%)*	*16 (1.15%)*
Other specialised construction activities	*-*	*-*	*8 (1.19%)*	*10 (1.07%)*	*10 (0.83%)*	*11 (0.79%)*	*11 (0.79%)*	*13 (0.93%)*	*12 (0.86%)*	*10 (0.72%)*	*23 (1.65%)*
Construction of buildings	1 (1.92%)	5 (1.39%)	7 (1.04%)	14 (1.50%)	16 (1.33%)	18 (1.29%)	19 (1.36%)	18 (1.29%)	20 (1.43%)	18 (1.29%)	40 (2.87%)
Development of building projects	*1 (1.92%)*	*-*	*-*	*-*	*1 (0.08%)*	*1 (0.07%)*	*1 (0.07%)*	*-*	*-*	*-*	*2 (0.14%)*
Construction of residential and non-residential buildings	*-*	*5 (1.39%)*	*7 (1.04%)*	*14 (1.50%)*	*15 (1.25%)*	*17 (1.22%)*	*18 (1.29%)*	*18 (1.29%)*	*20 (1.43%)*	*18 (1.29%)*	*38 (2.73%)*
Civil engineering	-	1 (0.28%)	3 (0.44%)	3 (0.32%)	6 (0.50%)	8 (0.57%)	7 (0.50%)	7 (0.50%)	6 (0.43%)	5 (0.36%)	13 (0.93%)
Repair and installation of machinery and equipment	1 (1.92%)	1 (0.28%)	3 (0.44%)	7 (0.75%)	6 (0.50%)	3 (0.22%)	4 (0.29%)	4 (0.29%)	4 (0.29%)	3 (0.22%)	9 (0.65%)
Public administration and defence; compulsory social security	-	-	-	1 (0.11%)	2 (0.17%)	3 (0.22%)	3 (0.22%)	4 (0.29%)	4 (0.29%)	3 (0.22%)	7 (0.50%)
Services to buildings and landscape activities	-	1 (0.28%)	-	2 (0.21%)	3 (0.25%)	3 (0.22%)	3 (0.22%)	2 (0.14%)	1 (0.07%)	2 (0.14%)	6 (0.43%)
Manufacture of coke and refined petroleum products	-	-	1 (0.15%)	-	-	1 (0.07%)	1 (0.07%)	2 (0.14%)	2 (0.14%)	2 (0.14%)	4 (0.29%)
Manufacture of other non-metallic mineral products	-	1 (0.28%)	-	2 (0.21%)	2 (0.17%)	2 (0.14%)	1 (0.07%)	-	-	-	4 (0.29%)
Manufacture of other transport equipment	-	2 (0.56%)	3 (0.44%)	3 (0.32%)	2 (0.17%)	1 (0.07%)	-	-	-	-	4 (0.29%)
Electricity, gas, steam and air conditioning supply	-	-	-	1 (0.11%)	3 (0.25%)	4 (0.29%)	3 (0.22%)	2 (0.14%)	1 (0.07%)	1 (0.07%)	4 (0.29%)
Mining of coal and lignite	-	2 (0.56%)	1 (0.15%)	1 (0.11%)	1 (0.08%)	-	-	-	-	-	3 (0.22%)
Manufacture of fabricated metal products, except machinery and equipment	-	-	-	-	-	1 (0.07%)	1 (0.07%)	2 (0.14%)	2 (0.14%)	3 (0.22%)	3 (0.22%)
Education	-	-	-	1 (0.11%)	1 (0.08%)	1 (0.07%)	1 (0.07%)	3 (0.22%)	3 (0.22%)	3 (0.22%)	3 (0.22%)
Sports activities and amusement and recreation activities	-	-	-	-	-	1 (0.07%)	1 (0.07%)	1 (0.07%)	3 (0.22%)	3 (0.22%)	3 (0.22%)
Mining support service activities	-	-	1 (0.15%)	1 (0.11%)	1 (0.08%)	1 (0.07%)	1 (0.07%)	1 (0.07%)	1 (0.07%)	1 (0.07%)	2 (0.14%)
Manufacture of food products	-	-	-	-	-	-	-	-	-	-	2 (0.14%)
Manufacture of wood and of products of wood and cork, except furniture; manufacture of articles of straw and plaiting materials	-	-	-	1 (0.11%)	-	-	-	-	-	-	2 (0.14%)
Warehousing and support activities for transportation	-	-	-	-	1 (0.08%)	1 (0.07%)	1 (0.07%)	1 (0.07%)	2 (0.14%)	2 (0.14%)	2 (0.14%)
Architectural and engineering activities; technical testing and analysis	-	-	1 (0.15%)	1 (0.11%)	1 (0.08%)	1 (0.07%)	1 (0.07%)	1 (0.07%)	1 (0.07%)	1 (0.07%)	2 (0.14%)
Human health activities	-	-	1 (0.15%)	1 (0.11%)	2 (0.17%)	2 (0.14%)	2 (0.14%)	2 (0.14%)	2 (0.14%)	2 (0.14%)	2 (0.14%)
Fishing and aquaculture	-	-	-	-	-	-	-	-	-	-	1 (0.07%)
Other mining and quarrying	-	-	1 (0.15%)	1 (0.11%)	1 (0.08%)	-	-	-	-	-	1 (0.07%)
Manufacture of electrical equipment	-	-	-	-	-	-	-	-	-	-	1 (0.07%)
Manufacture of machinery and equipment n.e.c.	-	-	-	-	-	1 (0.07%)	1 (0.07%)	1 (0.07%)	1 (0.07%)	1 (0.07%)	1 (0.07%)
Manufacture of motor vehicles, trailers and semi-trailers	-	-	-	-	-	-	-	-	-	-	1 (0.07%)
Manufacture of furniture	-	1 (0.28%)	-	-	-	-	-	-	-	-	1 (0.07%)
Other manufacturing	-	-	-	-	1 (0.08%)	1 (0.07%)	1 (0.07%)	1 (0.07%)	1 (0.07%)	-	1 (0.07%)
Sewerage	-	-	-	-	1 (0.08%)	1 (0.07%)	1 (0.07%)	1 (0.07%)	1 (0.07%)	1 (0.07%)	1 (0.07%)
Wholesale trade, except of motor vehicles and motorcycles	-	-	-	-	-	-	-	-	-	1 (0.07%)	1 (0.07%)
Land transport and transport via pipelines	-	-	-	-	1 (0.08%)	1 (0.07%)	1 (0.07%)	1 (0.07%)	1 (0.07%)	1 (0.07%)	1 (0.07%)
Accommodation	-	-	-	1 (0.11%)	1 (0.08%)	1 (0.07%)	1 (0.07%)	1 (0.07%)	1 (0.07%)	1 (0.07%)	1 (0.07%)
Telecommunications	-	-	-	1 (0.11%)	1 (0.08%)	1 (0.07%)	1 (0.07%)	1 (0.07%)	-	-	1 (0.07%)
Other professional, scientific and technical activities	-	-	-	-	-	1 (0.07%)	-	-	-	-	1 (0.07%)
Social work activities without accommodation	-	-	-	-	-	-	-	1 (0.07%)	1 (0.07%)	1 (0.07%)	1 (0.07%)
Other personal service activities	-	1 (0.28%)	1 (0.15%)	1 (0.11%)	1 (0.08%)	1 (0.07%)	1 (0.07%)	1 (0.07%)	1 (0.07%)	1 (0.07%)	1 (0.07%)

^a^The data presented for each industry in the “Overall” column correspond to the number of subjects over 18 years old having worked in a silica-exposed occupation in the sector concerned at least once between 1965 and 2010

Among women (*n* = 1548), exposure to crystalline silica concerned 9 subjects (0.6%) between 1965 and 2010. The sectors of activity found were: manufacture of other non-metallic mineral products (*n* = 2; 0.13%), manufacture of basic metals (*n* = 2; 0.13%), public administration, defence and compulsory social security (*n* = 2; 0.13%), public administration and defence; compulsory social security (*n* = 1; 0.06%), manufacture of computer, electronic and optical products (*n* = 1; 0.06%), specialised construction activities (*n* = 1; 0.06%), social work activities without accommodation (*n* = 1; 0.06%). The majority of exposures took place between 1980 and 2010.

## Discussion

This general population study described the proportions of subjects working in sectors of activity exposed to crystalline silica over a period of 45 years and highlighted a decrease in exposure to crystalline silica since the 1980s. This phenomenon had been highlighted in France by Delabre et al. (6.2% in 1982 against 4.1% in 2007 for the whole French population) [5]. The proportion of subjects exposed in 2010 remains significant with 4.4% of the total sample exposed and 9% in men only.

The analysis among men shows a high proportion of exposed workers in the construction activities (particularly in the specialised construction activities including electrical and plumbing installation activities), which is also highlighted by Delabre et al. who found 628,000 subjects in the construction sector in 2017 in France, i.e. 64% of all exposed subjects [5]. Abroad, this predominance of the construction sector is also found, notably in Sweden, where Gustavsson et al. estimated that in 2013, approximately 50% of the subjects exposed to crystalline silica worked in the construction sector [9]. There was also a very high proportion of workers in the manufacture of basic metals, which peaked between 1975 and 1985 (5.48% and 5.5% respectively) and then gradually decreased to 3.01% in 2010. This result can be partly explained by the fact that part of our sample lives in the metropolitan area of Dunkirk, which is a zone particularly rich in metallurgical industries. It should nevertheless be noted that the industrial sector, and in particular the steel industry, was the 3rd most frequent sector of activity in 2017 for exposure to silica according to Delabre et al. [5].

In our sample, exposure was mainly found among men (only 9 out of 1548 women had been exposed to silica during their career in our study). Here again, this phenomenon was also reported by Delabre et al. [5] (93% of the subjects exposed to silica in 2017 were men) but also outside France, notably in Australia [10] (10.2% exposed among men versus 1.2% exposed among women in a sample of 4993 Australians) and Sweden [9] (about 90% of exposed subjects were men in the 2013 census data). As the number of women exposed here is very low, the interest of comparing their sectors of activity to those of men is limited, as each sector only concerns 1 to 2 subjects at most.

One of the major strengths of our study is the presentation of data on the sectors of activity that expose to crystalline silica over the entire career of subjects randomly selected from the general population. The application of the Matgéné Silice JEM on career history allowed a description of these sectors over 45 years, starting in 1965, with a presentation of the proportions per 5-year period, making it possible to follow the evolution of each exposing sector over time and consequently to provide original information, not limited by the population census periods used in other studies [5, 9]. The drawback of our methodology in relation to the use of census data is the limitation of the number of subjects and the geographical scope. Indeed, we included subjects from the general population residing only in Lille and Dunkirk (a city with a relatively high level of industrial activity), which provided a good representation of the population of northern France, but, as we have shown, we find similar results to those obtained in studies with larger sample sizes, based on the proportion of exposed subjects alone, which shows the value of the additional information provided by our study (proportions of exposed subjects by sector of activity at different times during their career, from 1965 onwards) [5]. Another limitation is the transversal design, which does not allow us to eliminate the cohort and age effects, which may lead to a survival bias. Our cross-sectional study is limited to subjects aged between 40 and 65 at the time of the study, so there are few subjects who worked in 1965 and 1970 and the results must be interpreted with caution for these years, given the small numbers involved. The 40–65 age group was chosen in the ELISABET study, from which the sample was drawn, partly in order to assess the respiratory effects of repeated occupational and environmental exposure, with the lower limit at 40, and partly to limit the effects of greater geographical mobility after retirement, which may depend on health status and socio-economic level, with the upper limit at 65. Nevertheless, the death rates remain low at these ages. Finally, the use of the career history collected in a cross-sectional manner may have introduced a recall bias. This bias should be qualified by the fact that the subject was asked about his or her career as a whole and not about the presence of silica exposure, which was assessed afterwards.

This study shows that the proportion of workers exposed to crystalline silica has been decreasing since the 1980s, but still reaches significant levels, particularly in the construction sector, making occupational exposure to crystalline silica and its health consequences an ongoing issue. Our results also confirm the importance of collecting and tracing workers’ occupational careers as part of their occupational health monitoring, since the frequency of a given occupational exposure may vary over time on a population scale, as we have shown here for crystalline silica, all the more so if these exposures are at risk of causing pathologies with a long latency period.

## Data Availability

No datasets were generated or analysed during the current study.

## Abbreviations

JEM: Job-exposure matrix
ELISABET: Enquête Littoral Souffle Air Biologie Environnement
NACE: Statistical Classification of Economic Activities in the European Community revision 2
